# 3-{[5-(4-Chloro­phen­yl)-3-methyl-1*H*-pyrazol-1-yl]meth­yl}-4-*m*-tolyl-1*H*-1,2,4-triazole-5(4*H*)-thione

**DOI:** 10.1107/S1600536813013494

**Published:** 2013-05-22

**Authors:** Muhammad A. Farrukh, Maqsood Ahmed, Shaaban K. Mohamed, Adel A. Marzouk, Samir M. El-Moghazy

**Affiliations:** a Department of Chemistry, Government College University, Lahore 54000, Pakistan; b Chemistry and Environmental Division, Manchester Metropolitan University, Manchester, M1 5GD, England; c Chemistry Department, Faculty of Science, Minia University, El-Minia, Egypt; d Pharmaceutical Chemistry Department, Faculty of Pharmacy, Al Azhar University, Egypt; e Faculty of Pharmacy, Pharmaceutical Chemistry Department, Cairo University, Cairo, Egypt

## Abstract

In the title compound, C_20_H_18_ClN_5_S, the toluene and triazole rings are oriented almost perpendicular to each other, making a dihedral angle of 89.97 (9)°, whereas the dihedral angle between cholorophenyl and pyrazole rings is 54.57 (11)°. In the crystal, pairs of N—H⋯N hydrogen bonds link the mol­ecules into inversion dimers. Weaker C—H⋯S and C—H⋯Cl inter­actions are also present.

## Related literature

For medicinal applications of 1, 2, 4-triazoles, see: Lipinski (1983 ▶); Ram & Vlietinck (1988 ▶); Akahoshi *et al.* (1998 ▶); Young *et al.* (2001 ▶); Ouyang *et al.* (2005 ▶); Dolzhenko *et al.* (2007 ▶). For general background to the coordination chemistry of triazoles, see: Mishra *et al.* (1989 ▶); Klingele & Brooker (2003 ▶); Beckmann & Brooker (2003 ▶); Ferrer *et al.* (2004 ▶); Castineiras & Garcia-Santos (2008 ▶).
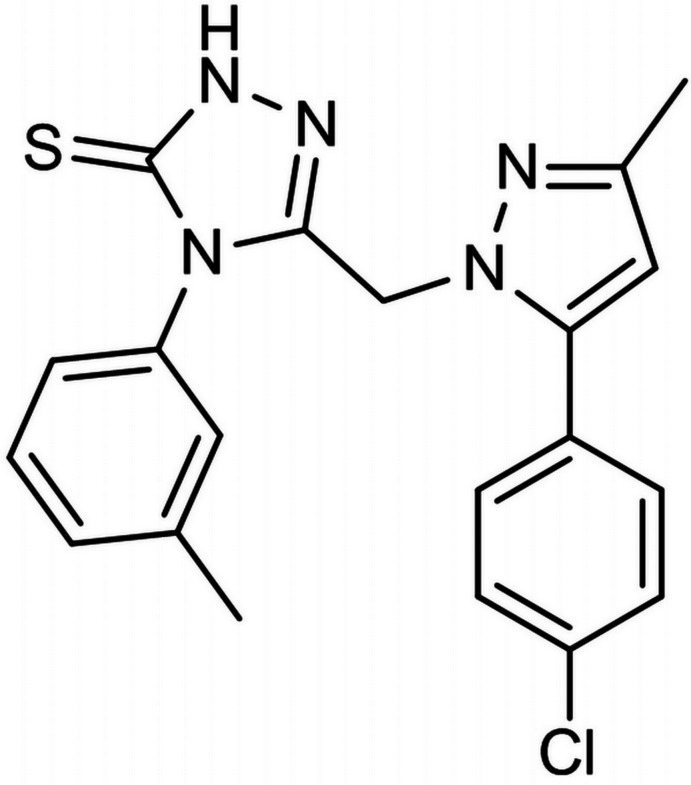


## Experimental

### 

#### Crystal data

C_20_H_18_ClN_5_S
*M* *_r_* = 395.90
Monoclinic, 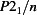

*a* = 8.328 (5) Å
*b* = 16.407 (5) Å
*c* = 14.759 (5) Å
β = 99.509 (5)°
*V* = 1988.9 (15) Å^3^
*Z* = 4
Mo *K*α radiation
μ = 0.31 mm^−1^
*T* = 296 K
0.61 × 0.53 × 0.52 mm

#### Data collection

Bruker APEXII CCD detector diffractometer
Absorption correction: analytical {*SADABS*; Bruker, 2009 ▶) *T*_min_ = 0.833, *T*_max_ = 0.855
38237 measured reflections
3914 independent reflections
3207 reflections with *I* > 2σ(*I*)
*R*_int_ = 0.031

#### Refinement

*R*[*F* ^2^ > 2σ(*F* ^2^)] = 0.038
*wR*(*F* ^2^) = 0.112
*S* = 1.04
3914 reflections
250 parameters
H atoms treated by a mixture of independent and constrained refinement
Δρ_max_ = 0.23 e Å^−3^
Δρ_min_ = −0.26 e Å^−3^

### 

Data collection: *APEX2* (Bruker, 2009 ▶); cell refinement: *SAINT* (Bruker, 2009 ▶); data reduction: *SAINT*; program(s) used to solve structure: *SIR92* (Altomare *et al.*, 1993 ▶); program(s) used to refine structure: *SHELXL97* (Sheldrick, 2008 ▶); molecular graphics: *ORTEP-3 for Windows* (Farrugia, 2012 ▶) and *Mercury* (Macrae *et al.*, 2008 ▶); software used to prepare material for publication: *publCIF* (Westrip, 2010 ▶).

## Supplementary Material

Crystal structure: contains datablock(s) I, global. DOI: 10.1107/S1600536813013494/gw2132sup1.cif

Structure factors: contains datablock(s) I. DOI: 10.1107/S1600536813013494/gw2132Isup2.hkl

Supplementary material file. DOI: 10.1107/S1600536813013494/gw2132Isup3.cml

Additional supplementary materials:  crystallographic information; 3D view; checkCIF report

## Figures and Tables

**Table 1:** Hydrogen-bond geometry (Å, °)

*D*—H⋯*A*	*D*—H	H⋯*A*	*D*⋯*A*	*D*—H⋯*A*
N4—H*N*4⋯N1^i^	0.88 (2)	2.02 (2)	2.888 (2)	166 (2)
C6—H6⋯S1^ii^	0.93	3.00	3.790 (3)	144
C20—H14⋯Cl1^iii^	0.93	2.98	3.537 (4)	120

Symmetry codes: (i) 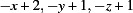; (ii) 
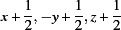
; (iii) 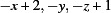.

**Fig. 1.**
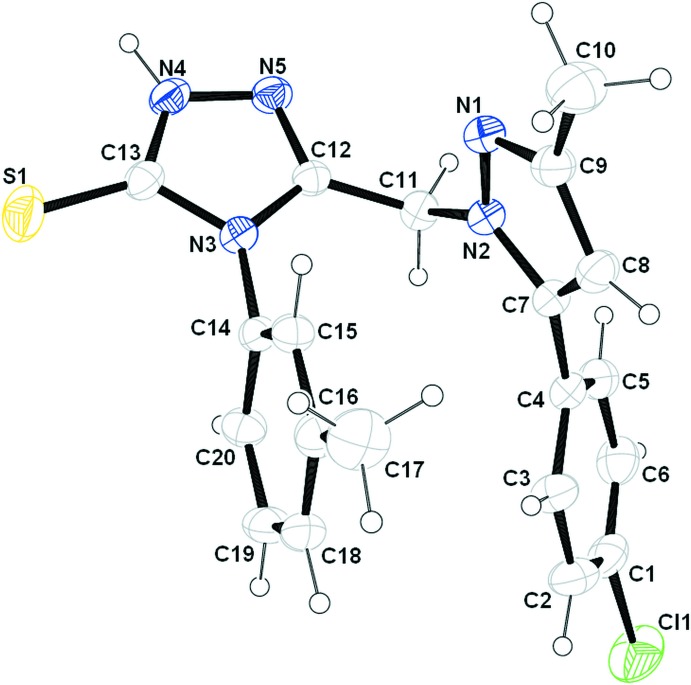
An ORTEPIII diagram of the molecule showing atom numbering scheme and thermal ellipsoids drawn at 30% probability level.

**Fig. 2.**
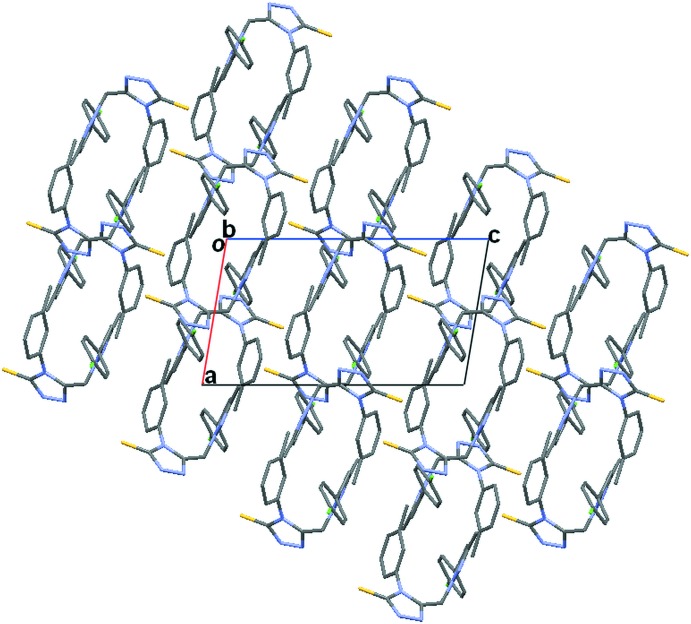
A view of the molecular packing along b axis.

**Fig. 3.**
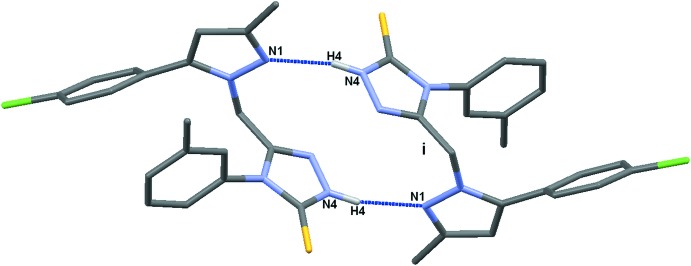
A dimer of molecules formed by the intermolecular N—H···N type hydrogen bonds. The H atoms not involved in any interaction have been omitted for the reason of clarity. [Symmetry code: (i) 2-x, 1-y, 1-z.]

**Fig. 4.**
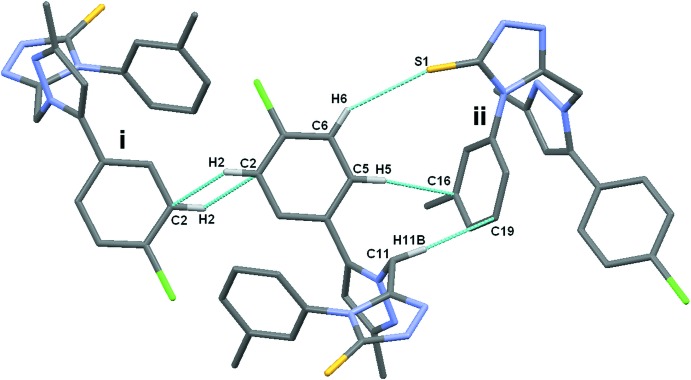
A view of the molecular packing showing weak intermolecular C—H···π and C—H···S interactions. [Symmetry codes: (i) 1-x, -y, 1-z; (ii) 1/2;+x, 1/2-y, 1/2+z.]

## supplementary crystallographic information

### Comment

1, 2, 4-Triazole compounds are used as reagents and ligands for the synthesis of biologically active compounds (Lipinski, 1983; Ram & Vlietinck, 1988; Akahoshi *et al.*, 1998; Young *et al.*, 2001; Ouyang *et al.*, 2005; Dolzhenko *et al.*, 2007) and metal complexes (Klingele & Brooker 2003; Beckmann & Brooker 2003; Mishra *et al.*, 1989; Ferrer *et al.*, 2004; Castineiras & Garcia-Santos, 2008). Further to our study on synthesis of bioactive heterocyclic compounds we herein report the synthesis and crystal structure of the title compound.

There is one molecule in the asymmetric unit and four molecules in the unit cell. 1, 2, 4 Triazole ring is oriented almost perpendicular to the toluene ring and the torsion angle between the two rings is 92.6 (2) ° as calculated on the basis of C13—N3—C14—C20 atoms. The diazole ring and the adjacent cholorobenzene ring adopt a twisted geometry and the torsion angle between them is -127.2 (2) ° as calculated using C3—C4—C7—N2 atoms. The molecular assembly is mainly built upon a reciprocal pair of intermolecular N4—H4···N1 type hydrogen bonds between two adjacent molecules which result in the formation of a molecular dimer. The D—H···A distance in each case is 2.02 Å. There are also some C—H···π interactions, a C—H···S interaction and a C—H···Cl interaction which help to stabilize the molecular assembly. The S1 atom accepts the H6 atom from C6 to form a C—H···S type weaker hydrogen bond at a distance of 2.998 Å with a D—H···A angle of 143.87°. Similarly the Cl1 atom accepts the H14 atom from C20 to form a C—H···Cl type weaker hydrogen bond at a distance of 2.978 Å with a D—H···A angle of 120.0°.

### Experimental

A solution of 2 N sodium hydroxide solution (2 ml) was added in dropwise to a solution of 5 mmol (2.07 g) 2-{[5-(4-chlorophenyl)-3-methyl-1*H*-pyrazol-1-yl]acetyl}-*N*-(3-methylphenyl)hydrazinecarbothioamide in 50 ml e thanol. The reaction mixture was then refluxed for 7 h, cooled and filtered. The filtrate was acidified with 2 N hydrochloric acid. The separated solid was collected, washed, and crystallized from ethanol in an excellent yield (92%). Single crystals suitable for X-ray diffraction were obtained from slow evaporation solution of the title compound in ethanol at room temperature (*M*.p. 433–435 K).

### Refinement

The methyl group H atoms were geometrically placed at idealized positions and refined using a riding model with d(C—H) = 0.96 Å and *U*_iso_= 1.5Ueq(C). The H atom on N atom was located in the difference map and was refined isotropically. All other H atoms bound to C were located in the difference map but were refined using a riding model with d(C—H) = 0.93 Å for aromatic and 0.97 Å for CH_2_ group with U*_iso_*(H) = 1.2*U*_eq_(C).

### Crystal data

**Table:**

C_20_H_18_ClN_5_S	*F*(000) = 824
*M**_r_* = 395.90	*D*_x_ = 1.322 Mg m^−^^3^
Monoclinic, *P*2_1_/*n*	Mo *K*α radiation, λ = 0.71073 Å
Hall symbol: -P 2yn	Cell parameters from 143 reflections
*a* = 8.328 (5) Å	θ = 1.9–26.5°
*b* = 16.407 (5) Å	µ = 0.31 mm^−^^1^
*c* = 14.759 (5) Å	*T* = 296 K
β = 99.509 (5)°	Block, colourless
*V* = 1988.9 (15) Å^3^	0.61 × 0.53 × 0.52 mm
*Z* = 4	

### Data collection

**Table:**

Bruker APEXII CCD detector diffractometer	3914 independent reflections
Radiation source: fine-focus sealed tube	3207 reflections with *I* > 2σ(*I*)
Graphite monochromator	*R*_int_ = 0.031
ω and φ scans	θ_max_ = 26.0°, θ_min_ = 1.9°
Absorption correction: analytical {*SADABS*; Bruker, 2009)	*h* = −10→10
*T*_min_ = 0.833, *T*_max_ = 0.855	*k* = −20→20
38237 measured reflections	*l* = −18→18

### Refinement

**Table:**

Refinement on *F*^2^	Primary atom site location: structure-invariant direct methods
Least-squares matrix: full	Secondary atom site location: difference Fourier map
*R*[*F*^2^ > 2σ(*F*^2^)] = 0.038	Hydrogen site location: inferred from neighbouring sites
*wR*(*F*^2^) = 0.112	H atoms treated by a mixture of independent and constrained refinement
*S* = 1.04	*w* = 1/[σ^2^(*F*_o_^2^) + (0.0474*P*)^2^ + 0.8662*P*] where *P* = (*F*_o_^2^ + 2*F*_c_^2^)/3
3914 reflections	(Δ/σ)_max_ = 0.008
250 parameters	Δρ_max_ = 0.23 e Å^−^^3^
0 restraints	Δρ_min_ = −0.26 e Å^−^^3^

### Special details

**Table:**

Geometry. All e.s.d.'s (except the e.s.d. in the dihedral angle between two l.s. planes) are estimated using the full covariance matrix. The cell e.s.d.'s are taken into account individually in the estimation of e.s.d.'s in distances, angles and torsion angles; correlations between e.s.d.'s in cell parameters are only used when they are defined by crystal symmetry. An approximate (isotropic) treatment of cell e.s.d.'s is used for estimating e.s.d.'s involving l.s. planes.
Refinement. Refinement of *F*^2^ against ALL reflections. The weighted *R*-factor *wR* and goodness of fit *S* are based on *F*^2^, conventional *R*-factors *R* are based on *F*, with *F* set to zero for negative *F*^2^. The threshold expression of *F*^2^ > σ(*F*^2^) is used only for calculating *R*-factors(gt) *etc*. and is not relevant to the choice of reflections for refinement. *R*-factors based on *F*^2^ are statistically about twice as large as those based on *F*, and *R*- factors based on ALL data will be even larger.

### Fractional atomic coordinates and isotropic or equivalent isotropic displacement parameters (Å^2^)

**Table:**

	*x*	*y*	*z*	*U*_iso_*/*U*_eq_	
S1	0.89331 (8)	0.45028 (4)	0.23277 (4)	0.0738 (2)	
Cl1	0.87201 (11)	−0.10903 (4)	0.56431 (6)	0.1018 (3)	
N2	0.83576 (17)	0.30784 (8)	0.58288 (10)	0.0420 (3)	
N3	0.91296 (16)	0.36199 (8)	0.39136 (10)	0.0402 (3)	
N1	0.76919 (18)	0.38104 (9)	0.59822 (10)	0.0460 (3)	
N4	1.08074 (19)	0.46017 (9)	0.40066 (11)	0.0474 (4)	
N5	1.10761 (18)	0.42403 (9)	0.48545 (11)	0.0468 (4)	
C12	1.00462 (19)	0.36448 (10)	0.47753 (11)	0.0396 (4)	
C14	0.78212 (19)	0.30653 (10)	0.35998 (11)	0.0388 (4)	
C15	0.6269 (2)	0.32639 (10)	0.37306 (12)	0.0438 (4)	
H19	0.6080	0.3758	0.4003	0.053*	
C4	0.7762 (2)	0.15886 (10)	0.58340 (12)	0.0453 (4)	
C13	0.9620 (2)	0.42534 (10)	0.34117 (13)	0.0457 (4)	
C11	0.9930 (2)	0.30522 (11)	0.55227 (12)	0.0457 (4)	
H11A	1.0112	0.2507	0.5307	0.055*	
H11B	1.0780	0.3168	0.6040	0.055*	
C20	0.8140 (2)	0.23434 (11)	0.31944 (13)	0.0492 (4)	
H14	0.9187	0.2219	0.3097	0.059*	
C9	0.6232 (2)	0.36378 (11)	0.61886 (13)	0.0506 (4)	
C16	0.4988 (2)	0.27320 (12)	0.34583 (13)	0.0491 (4)	
C18	0.5317 (2)	0.20020 (12)	0.30670 (13)	0.0548 (5)	
H16	0.4474	0.1634	0.2889	0.066*	
C7	0.7349 (2)	0.24523 (10)	0.59382 (12)	0.0445 (4)	
C6	0.9473 (3)	0.04243 (13)	0.62598 (15)	0.0643 (6)	
H6	1.0406	0.0195	0.6597	0.077*	
C8	0.5974 (2)	0.27957 (11)	0.61661 (14)	0.0529 (5)	
H8	0.5050	0.2523	0.6283	0.063*	
C5	0.9165 (3)	0.12459 (12)	0.63229 (13)	0.0564 (5)	
H5	0.9907	0.1574	0.6698	0.068*	
C19	0.6871 (3)	0.18060 (12)	0.29343 (14)	0.0574 (5)	
H15	0.7066	0.1310	0.2668	0.069*	
C3	0.6710 (2)	0.10971 (11)	0.52508 (14)	0.0551 (5)	
H3	0.5775	0.1321	0.4910	0.066*	
C2	0.7034 (3)	0.02772 (12)	0.51691 (16)	0.0640 (6)	
H2	0.6342	−0.0049	0.4763	0.077*	
C1	0.8389 (3)	−0.00466 (12)	0.56953 (16)	0.0605 (5)	
C17	0.3290 (3)	0.29548 (17)	0.3590 (2)	0.0794 (7)	
H17A	0.2554	0.2525	0.3357	0.119*	
H17B	0.2966	0.3450	0.3264	0.119*	
H17C	0.3266	0.3032	0.4232	0.119*	
C10	0.5133 (3)	0.43051 (14)	0.64055 (19)	0.0749 (7)	
H10A	0.5713	0.4813	0.6453	0.112*	
H10B	0.4776	0.4189	0.6978	0.112*	
H10C	0.4206	0.4341	0.5925	0.112*	
HN4	1.130 (2)	0.5065 (14)	0.3917 (14)	0.056 (6)*	

### Atomic displacement parameters (Å^2^)

**Table:**

	*U*^11^	*U*^22^	*U*^33^	*U*^12^	*U*^13^	*U*^23^
S1	0.0868 (4)	0.0712 (4)	0.0609 (3)	−0.0027 (3)	0.0045 (3)	0.0243 (3)
Cl1	0.1391 (7)	0.0416 (3)	0.1352 (7)	0.0167 (3)	0.0533 (5)	0.0124 (3)
N2	0.0469 (8)	0.0364 (7)	0.0430 (8)	−0.0039 (6)	0.0086 (6)	−0.0001 (6)
N3	0.0405 (7)	0.0361 (7)	0.0437 (7)	−0.0015 (6)	0.0060 (6)	0.0015 (6)
N1	0.0528 (9)	0.0380 (7)	0.0480 (8)	−0.0046 (6)	0.0106 (7)	−0.0024 (6)
N4	0.0500 (8)	0.0369 (8)	0.0580 (9)	−0.0073 (7)	0.0169 (7)	−0.0003 (7)
N5	0.0456 (8)	0.0439 (8)	0.0518 (9)	−0.0070 (6)	0.0110 (7)	−0.0043 (7)
C12	0.0371 (8)	0.0379 (8)	0.0443 (9)	0.0000 (7)	0.0081 (7)	−0.0030 (7)
C14	0.0411 (8)	0.0350 (8)	0.0391 (8)	−0.0011 (6)	0.0033 (6)	0.0012 (6)
C15	0.0457 (9)	0.0398 (9)	0.0463 (9)	0.0016 (7)	0.0083 (7)	−0.0041 (7)
C4	0.0549 (10)	0.0377 (9)	0.0444 (9)	−0.0048 (8)	0.0117 (8)	0.0039 (7)
C13	0.0480 (9)	0.0367 (8)	0.0545 (10)	0.0030 (7)	0.0147 (8)	0.0030 (7)
C11	0.0425 (9)	0.0477 (10)	0.0461 (10)	−0.0012 (7)	0.0050 (7)	0.0030 (7)
C20	0.0498 (10)	0.0450 (10)	0.0523 (10)	0.0062 (8)	0.0071 (8)	−0.0048 (8)
C9	0.0540 (10)	0.0464 (10)	0.0536 (11)	−0.0045 (8)	0.0151 (8)	−0.0054 (8)
C16	0.0445 (9)	0.0555 (11)	0.0470 (10)	−0.0047 (8)	0.0067 (8)	0.0019 (8)
C18	0.0605 (11)	0.0508 (11)	0.0496 (10)	−0.0170 (9)	−0.0005 (9)	−0.0017 (8)
C7	0.0523 (10)	0.0402 (9)	0.0409 (9)	−0.0078 (8)	0.0078 (7)	0.0016 (7)
C6	0.0717 (13)	0.0574 (12)	0.0630 (13)	0.0118 (11)	0.0093 (11)	0.0171 (10)
C8	0.0533 (10)	0.0478 (10)	0.0609 (11)	−0.0107 (8)	0.0192 (9)	−0.0012 (9)
C5	0.0649 (12)	0.0531 (11)	0.0485 (10)	−0.0038 (9)	0.0014 (9)	0.0046 (9)
C19	0.0731 (13)	0.0392 (9)	0.0575 (11)	−0.0003 (9)	0.0036 (10)	−0.0106 (8)
C3	0.0544 (11)	0.0431 (10)	0.0653 (12)	−0.0049 (8)	0.0026 (9)	0.0015 (9)
C2	0.0693 (13)	0.0439 (11)	0.0789 (15)	−0.0115 (10)	0.0124 (11)	−0.0084 (10)
C1	0.0747 (14)	0.0390 (10)	0.0729 (14)	0.0043 (9)	0.0268 (11)	0.0095 (9)
C17	0.0482 (12)	0.0956 (18)	0.0968 (18)	−0.0064 (12)	0.0191 (12)	−0.0075 (15)
C10	0.0723 (14)	0.0598 (13)	0.0986 (19)	0.0036 (11)	0.0323 (13)	−0.0114 (12)

### Geometric parameters (Å, º)

**Table:**

S1—C13	1.659 (2)	C20—H14	0.9300
Cl1—C1	1.738 (2)	C9—C8	1.398 (3)
N2—C7	1.353 (2)	C9—C10	1.495 (3)
N2—N1	1.357 (2)	C16—C18	1.377 (3)
N2—C11	1.455 (2)	C16—C17	1.504 (3)
N3—C12	1.372 (2)	C18—C19	1.379 (3)
N3—C13	1.377 (2)	C18—H16	0.9300
N3—C14	1.436 (2)	C7—C8	1.367 (3)
N1—C9	1.332 (2)	C6—C1	1.363 (3)
N4—C13	1.337 (2)	C6—C5	1.378 (3)
N4—N5	1.369 (2)	C6—H6	0.9300
N4—HN4	0.89 (2)	C8—H8	0.9300
N5—C12	1.293 (2)	C5—H5	0.9300
C12—C11	1.486 (2)	C19—H15	0.9300
C14—C20	1.373 (2)	C3—C2	1.381 (3)
C14—C15	1.377 (2)	C3—H3	0.9300
C15—C16	1.385 (2)	C2—C1	1.367 (3)
C15—H19	0.9300	C2—H2	0.9300
C4—C3	1.382 (3)	C17—H17A	0.9600
C4—C5	1.387 (3)	C17—H17B	0.9600
C4—C7	1.472 (2)	C17—H17C	0.9600
C11—H11A	0.9700	C10—H10A	0.9600
C11—H11B	0.9700	C10—H10B	0.9600
C20—C19	1.381 (3)	C10—H10C	0.9600
			
C7—N2—N1	111.89 (14)	C15—C16—C17	120.32 (19)
C7—N2—C11	128.53 (15)	C16—C18—C19	121.26 (17)
N1—N2—C11	119.43 (13)	C16—C18—H16	119.4
C12—N3—C13	107.90 (14)	C19—C18—H16	119.4
C12—N3—C14	126.46 (14)	N2—C7—C8	106.16 (16)
C13—N3—C14	125.54 (14)	N2—C7—C4	123.88 (16)
C9—N1—N2	105.27 (14)	C8—C7—C4	129.96 (16)
C13—N4—N5	113.98 (15)	C1—C6—C5	118.9 (2)
C13—N4—HN4	125.5 (13)	C1—C6—H6	120.5
N5—N4—HN4	119.9 (13)	C5—C6—H6	120.5
C12—N5—N4	103.82 (14)	C7—C8—C9	106.34 (16)
N5—C12—N3	111.46 (15)	C7—C8—H8	126.8
N5—C12—C11	123.46 (16)	C9—C8—H8	126.8
N3—C12—C11	125.05 (15)	C6—C5—C4	120.8 (2)
C20—C14—C15	121.24 (16)	C6—C5—H5	119.6
C20—C14—N3	119.76 (15)	C4—C5—H5	119.6
C15—C14—N3	118.99 (15)	C18—C19—C20	120.32 (18)
C14—C15—C16	120.39 (16)	C18—C19—H15	119.8
C14—C15—H19	119.8	C20—C19—H15	119.8
C16—C15—H19	119.8	C4—C3—C2	120.66 (19)
C3—C4—C5	118.72 (18)	C4—C3—H3	119.7
C3—C4—C7	119.44 (17)	C2—C3—H3	119.7
C5—C4—C7	121.82 (17)	C1—C2—C3	118.9 (2)
N4—C13—N3	102.84 (15)	C1—C2—H2	120.5
N4—C13—S1	128.86 (14)	C3—C2—H2	120.5
N3—C13—S1	128.28 (14)	C6—C1—C2	121.89 (19)
N2—C11—C12	112.56 (14)	C6—C1—Cl1	119.44 (18)
N2—C11—H11A	109.1	C2—C1—Cl1	118.67 (18)
C12—C11—H11A	109.1	C16—C17—H17A	109.5
N2—C11—H11B	109.1	C16—C17—H17B	109.5
C12—C11—H11B	109.1	H17A—C17—H17B	109.5
H11A—C11—H11B	107.8	C16—C17—H17C	109.5
C14—C20—C19	118.55 (17)	H17A—C17—H17C	109.5
C14—C20—H14	120.7	H17B—C17—H17C	109.5
C19—C20—H14	120.7	C9—C10—H10A	109.5
N1—C9—C8	110.33 (17)	C9—C10—H10B	109.5
N1—C9—C10	120.48 (18)	H10A—C10—H10B	109.5
C8—C9—C10	129.19 (19)	C9—C10—H10C	109.5
C18—C16—C15	118.22 (17)	H10A—C10—H10C	109.5
C18—C16—C17	121.46 (18)	H10B—C10—H10C	109.5
			
C7—N2—N1—C9	−0.24 (19)	C14—C15—C16—C18	0.8 (3)
C11—N2—N1—C9	175.82 (15)	C14—C15—C16—C17	−179.16 (19)
C13—N4—N5—C12	1.10 (19)	C15—C16—C18—C19	−1.0 (3)
N4—N5—C12—N3	−0.47 (18)	C17—C16—C18—C19	178.9 (2)
N4—N5—C12—C11	177.59 (15)	N1—N2—C7—C8	0.4 (2)
C13—N3—C12—N5	−0.25 (19)	C11—N2—C7—C8	−175.26 (16)
C14—N3—C12—N5	−176.79 (15)	N1—N2—C7—C4	−178.73 (15)
C13—N3—C12—C11	−178.27 (15)	C11—N2—C7—C4	5.7 (3)
C14—N3—C12—C11	5.2 (3)	C3—C4—C7—N2	−127.2 (2)
C12—N3—C14—C20	−91.4 (2)	C5—C4—C7—N2	54.4 (3)
C13—N3—C14—C20	92.6 (2)	C3—C4—C7—C8	54.0 (3)
C12—N3—C14—C15	87.3 (2)	C5—C4—C7—C8	−124.4 (2)
C13—N3—C14—C15	−88.7 (2)	N2—C7—C8—C9	−0.3 (2)
C20—C14—C15—C16	0.4 (3)	C4—C7—C8—C9	178.69 (18)
N3—C14—C15—C16	−178.24 (16)	N1—C9—C8—C7	0.2 (2)
N5—N4—C13—N3	−1.22 (19)	C10—C9—C8—C7	−179.6 (2)
N5—N4—C13—S1	−179.74 (13)	C1—C6—C5—C4	1.1 (3)
C12—N3—C13—N4	0.86 (17)	C3—C4—C5—C6	−2.7 (3)
C14—N3—C13—N4	177.44 (14)	C7—C4—C5—C6	175.69 (18)
C12—N3—C13—S1	179.39 (14)	C16—C18—C19—C20	0.0 (3)
C14—N3—C13—S1	−4.0 (3)	C14—C20—C19—C18	1.1 (3)
C7—N2—C11—C12	129.99 (18)	C5—C4—C3—C2	1.2 (3)
N1—N2—C11—C12	−45.3 (2)	C7—C4—C3—C2	−177.28 (18)
N5—C12—C11—N2	117.51 (18)	C4—C3—C2—C1	1.9 (3)
N3—C12—C11—N2	−64.7 (2)	C5—C6—C1—C2	2.1 (3)
C15—C14—C20—C19	−1.3 (3)	C5—C6—C1—Cl1	−177.48 (16)
N3—C14—C20—C19	177.29 (16)	C3—C2—C1—C6	−3.6 (3)
N2—N1—C9—C8	0.0 (2)	C3—C2—C1—Cl1	175.96 (17)
N2—N1—C9—C10	179.80 (19)		

### Hydrogen-bond geometry (Å, º)

**Table:**

*D*—H···*A*	*D*—H	H···*A*	*D*···*A*	*D*—H···*A*
N4—H*N*4···N1^i^	0.88 (2)	2.02 (2)	2.888 (2)	166 (2)
C5—H5···C16^ii^	0.93	2.83	3.536 (3)	134
C6—H6···S1^ii^	0.93	3.00	3.790 (3)	144
C2—H2···C2^iii^	0.93	2.85	3.464 (4)	124
C20—H14···Cl1^iv^	0.93	2.98	3.537 (4)	120

Symmetry codes: (i) −*x*+2, −*y*+1, −*z*+1; (ii) *x*+1/2, −*y*+1/2, *z*+1/2; (iii) −*x*+1, −*y*, −*z*+1; (iv) −*x*+2, −*y*, −*z*+1.
